# Learning-Directed Dynamic Voltage and Frequency Scaling Scheme with Adjustable Performance for Single-Core and Multi-Core Embedded and Mobile Systems †

**DOI:** 10.3390/s18093068

**Published:** 2018-09-12

**Authors:** Yen-Lin Chen, Ming-Feng Chang, Chao-Wei Yu, Xiu-Zhi Chen, Wen-Yew Liang

**Affiliations:** 1 Department of Computer Science and Information Engineering, National Taipei University of Technology, Taipei 10608, Taiwan; david741002@gmail.com (C.-W.Y.); leoshiou@gmail.com (X.-Z.C.); wyliang@mail.ntut.edu.tw (W.-Y.L.); 2 MediaTek Inc., Hsinchu 30078, Taiwan; winner121@gmail.com

**Keywords:** dynamic voltage and frequency scaling (DVFS), embedded systems, energy consumption, low-power software design, multicore computing systems, mobile devices

## Abstract

Dynamic voltage and frequency scaling (DVFS) is a well-known method for saving energy consumption. Several DVFS studies have applied learning-based methods to implement the DVFS prediction model instead of complicated mathematical models. This paper proposes a lightweight learning-directed DVFS method that involves using counter propagation networks to sense and classify the task behavior and predict the best voltage/frequency setting for the system. An intelligent adjustment mechanism for performance is also provided to users under various performance requirements. The comparative experimental results of the proposed algorithms and other competitive techniques are evaluated on the NVIDIA JETSON Tegra K1 multicore platform and Intel PXA270 embedded platforms. The results demonstrate that the learning-directed DVFS method can accurately predict the suitable central processing unit (CPU) frequency, given the runtime statistical information of a running program, and achieve an energy savings rate up to 42%. Through this method, users can easily achieve effective energy consumption and performance by specifying the factors of performance loss.

## 1. Introduction

Dynamic voltage and frequency scaling is a well-known method for reducing power consumption in modern consumer mobile devices. Dynamic power is consumed because of the switching of gates that cost major parts of power consumption dissipated in complementary metal-oxide-semiconductor (CMOS) circuits. DVFS can be easily implemented in a real-time system under the timing constraints, and the task can be executed with a lower CPU frequency and voltage with satisfactory performance that can reduce power dissipation. Video decoding is a common real-time application; the execution of the decoding process can be slowed to enable it to finish precisely at its deadline [1,2]. However, these DVFS techniques may not be suitable for general-purpose applications because they mostly entail assuming that the task arrival time, deadline, and workload are known in advance. In recent years, DVFS research has increasingly focused on general-purpose computing applications. The main concept of using the DVFS technique for general-purpose applications is to supply a minimal voltage and frequency when the CPU is in the idle mode. Thus, the CPU voltage and frequency for memory-bound applications can be scaled down. This paper proposes a machine-learning-based method that intelligently analyzes the behavior of CPU–memory relationships for non-real-time systems in order to select the optimally-fitted CPU computational speed. The fundamental concept of our approach is to transform a DVFS problem into a pattern classification problem with the task features. This paper used a neural network based method to perform this learning-based DVFS control algorithm.

A crucial problem of the learning model and mathematical model for the DVFS algorithm is time complexity. Rangan et al. [3] first asserted that operating system (OS) scheduler sampling intervals are on the millisecond time scale, whereas the computational requirements can vary on a nanosecond time scale because of CPU events such as cache misses. When the DVFS control algorithm is considerably complex, it requires excessive time to respond to such fine variations in program behavior. To solve this problem regarding the computational complexity of DVFS, this study proposes a learning-based approach based on the counter propagation networks (CPN), to simplify the DVFS control algorithm. The CPN is a fast and coarse approximation for vector mapping problems. It has been widely used to solve such problems because of its simplicity, easy training characteristics, and satisfactory statistical representation capability of the input environment. In this study, we apply the CPN approach to learn and classify the task behaviors into proper CPU frequencies and voltages.

In recent years, demand for high-performance embedded computing systems capable of performing multitasking operations has increased. Chip multiprocessors (CMPs) have emerged as a key technology for embedded computing demand. Excessive energy consumption in multicore computing systems has also become a crucial problem for handheld embedded systems. Thus, the proposed scheme solves DVFS problems for both single-core and multicore embedded computing systems. Because there is a trade-off between energy consumption and execution performance, for improving energy consumption, this study proposes a learning-directed algorithm to choose an appropriate CPU frequency to optimally minimize the energy consumption under various performance requirements.

This paper proposes a lightweight learning-based DVFS technique for single-core and multicore embedded systems. This technique was implemented and evaluated on the Linux operating system running on an Intel PXA27x embedded platform (Intel, Santa Clara, CA, USA) and NVIDIA Tegra K1 multicore platforms (NVIDIA, Santa Clara, CA, USA). For the experiments, several benchmarks with different behaviors were selected from MiBench [4] and ParMiBench [5] to demonstrate the performance of the proposed scheme. Real energy data were collected using a high-performance data acquisition instrument (DAQ). According to the experimental results, the proposed learning-directed DVFS technique can achieve an energy savings rate up to 42%.

The paper is structured as follows. Section 2 describes related works, and Section 3 presents the CPN that is used in the proposed learning-directed algorithm. In Section 4, the proposed learning-based DVFS algorithm is introduced and explained, and the training data are also discussed. Section 5 describes the implementation of the DVFS mechanism. The experimental results and a comparison with the standard Linux DVFS implementations are reported in Section 6. Finally, Section 7 draws conclusions.

## 2. Related Works

The CPU and memory are crucial computing resources for general-purpose operating systems. The DVFS algorithm adjusts the CPU frequency to reduce power consumption when the system is executing memory-bound jobs [6,7,8,9]. Choi et al. [6] proposed a DVFS method using workload decomposition, in which the system workload is decomposed into two parts: on-chip and off-chip parts. This decomposition is determined according to run-time statistics reported by the hardware performance counters. The on-chip workload signifies the clock cycles of the executed instruction spending in CPU operations, and the off-chip part represents the cycles of memory accesses. Poellabauer et al. [8] used cache miss rates as the indicator for memory access rates (MAR) and introduced a more reliable predictor of the next task execution times. Catania et al. [9] proposed an energy managing technique in wireless network-on-chip architectures. This method is performed by selectively turning off appropriate numbers of cycles and was assessed under several scenarios, obtaining 25% energy saving results without impact on performance metrics.

Subsequent studies [7,10] have proven that the minimum energy consumption of a system may not appear at the slowest operating speed of a system. Jejurikar and Gupta [10] defined the critical speed of a task as the clock rate with which a system can have the minimum energy consumption for that particular task. In our previous study [7], a memory-aware DVFS algorithm was also proposed according to this phenomenon. The assumption behind the algorithm is that reducing the frequency may not consistently induce lower energy consumption. The relationship between memory access behavior and critical speeds is used to predict an ideal frequency that tends to minimize energy consumption. However, some critical problems still exist in this CPU–memory DVFS method. Eyerman and Eeckhout [11] asserted that the limitation of estimated linear scaling is that it does not count the impact of multiple memory accesses overlapping in time on the off-chip part of the execution time, thus generating an inaccurate DVFS prediction. Because of these problems, the aim of this study was to discover a more efficient approach to automatically identifying the relationships between CPU and memory in order to predict a suitable voltage and frequency setting.

In [12], Kim proposed an online DVFS scheme for a real-time system based on the NBS model. Lahiri et al. [13] adapted an ANN method approach to the DVFS problem. The neural network in [13] is trained using the back-propagation network technique to predict the future computational load. The frequency can be generated according to the varying computational load. In Dhiman and Rosing [14], the online learning algorithm was proposed to estimate the most suitable voltage and frequency setting. This DVFS online learning algorithm was also implemented on an Intel PXA27x platform. We implemented the online learning method [14] for comparison with our proposed method in the single-core system. Moeng and Melhem [15] employed a decision tree algorithm to predict the frequency that minimizes the expected energy per user-instruction or energy per (user-instruction)^2^. Qingchen [16] proposed Deep Q-Learning model, which combined different DVFS technologies as a hybrid algorithm for power consumption reduction. By the defined Q-function to calculate the Q-value for each DVFS technology, the smallest Q-value is selected to adjust the voltage and frequency in the next hyperperiod. Jung and Pedram [17] presented a supervised-learning-based DVFS algorithm using the Bayesian classification technique, in which the algorithm learns to predict the system performance state on the basis of the predefined input features and then uses this predicted state to identify the optimal power management action from a precomputed policy lookup table. This DVFS approach [17] was also implemented in the NVIDIA Tegra K1 platform for comparison with the multicore DVFS approach in this study. Tesauro et al. [18] presented a reinforcement learning approach to control the power management policies that produce intelligent trade-offs between power and performance. Isci [19] classified the task characteristics into six phases according to MARs and proposed a global phase history table predictor to anticipate the future phase. However, in this study, the phase mapping onto DVFS mappings was not rigorous for the general-purpose computing applications of modern mobile systems.

Our previous research [20] proposed a simple DVFS prediction for a DVFS algorithm (P-DVFS). The prediction formula is

(1) $$f_{t}=\alpha \cdot f_{t-1}\frac{p_{exp}}{p_{t-1}},$$

where *f_t_* is the predicted frequency at time interval *t*, and *f_t_*_−1_ is the adjusted frequency at time interval *t* − 1. *p_exp_* is the performance factor that a user expects, and *p_t_*_−1_ is the performance factor executed at a frequency of *f_t_*_−1_, where *α* is a constant being greater than one, that defines the degree of the frequency adjustment. This formula increases the CPU frequency when *p_t_*_−1_ is lower than *p*_exp_ and decreases the CPU frequency when *p_t_*_−1_ is higher than *p*_exp_. P-DVFS uses a simple factor *p*_exp_/*p_t_*_−1_ to predict the CPU frequency.

**Figure 1:**
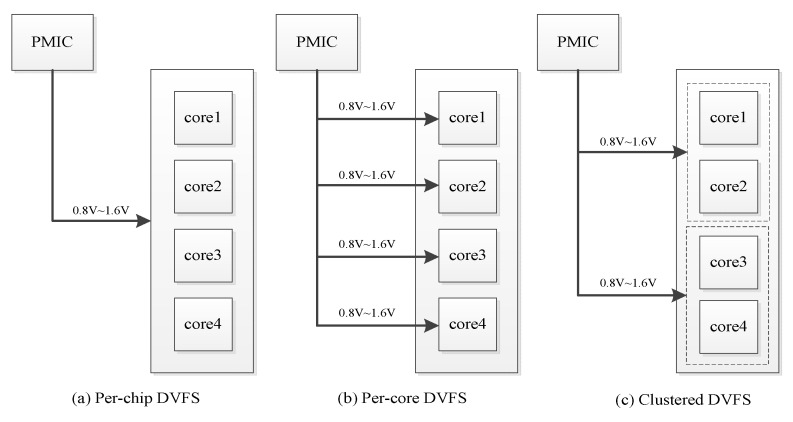
Four-core Multiprocessor system-on-chip (MPSoC) with various DVFS domains.

Presently, embedded systems—such as smartphones, tablet PCs, and handheld devices—are widely applied in daily life. As the applications of these devices become more complex, performance must be increased while energy consumption is maintained at acceptable levels, especially for handheld battery-supported devices. Multicore processors may achieve high performance under some energy constraints. A key element of the multicore DVFS technique is the use of one or more voltage regulators that deliver power to a circuit. DVFS for a multicore system can be performed at various levels of granularity: per-chip DVFS [21], per-core DVFS [1,22,23], and cluster DVFS [1,24,25]. In Figure 1, PMIC stands for Power Management Integrated Circuit. Per-chip DVFS (Figure 1a) uses the same voltage regulator to each core and each core uses the same voltage and frequency setting. Per-core DVFS (Figure 1b) uses different voltage regulators for each core and allows the core to simultaneously use different voltage and frequency settings. Cluster DVFS (Figure 1c) uses multiple on-chip regulators to drive a set of voltage/frequency islands.

This paper proposes a lightweight learning-directed DVFS technique called CPN-DVFS. For classifying the task behavior into suitable speed frequencies, the proposed CPN-DVFS was designed to apply to both single-core and multicore embedded systems. The purpose was to transform a DVFS problem into a classification problem with task characteristics. An Artificial Neural Network (ANN) method was employed to execute this learning control algorithm. In our previous study [21], the CPN learning-directed concept was preliminarily adopted for computational time prediction on single-core platforms. In this study, we improve the CPN learning-directed concept presented in this previous study, and further propose a novel CPN-DVFS scheme to modern multicore embedded platforms, and design a new experimental environment on multicore platforms. For the comparative performance evaluation with the CPN-DVFS scheme, the DVFS for the single-core and multicore environments in previous studies [14,17,21] were also implemented in the experimental platforms. The previous studies [14,17] performed learning-based DVFS and eDVFS [21] based on the calculated model of the CPU stall time and power consumption to minimize total energy consumption in multicore platforms. The main contributions of our study are as follows: (1) an efficient lightweight learning-directed DVFS algorithm is proposed for both single-core and multicore embedded systems; (2) users are allowed to define the acceptable level of performance; (3) it achieves power savings of up to 42%.

## 3. Counter Propagation Networks

This section introduces the CPN that is used in the proposed learning-directed algorithm.

### 3.1. Counter Propagation Networks

**Figure 2:**
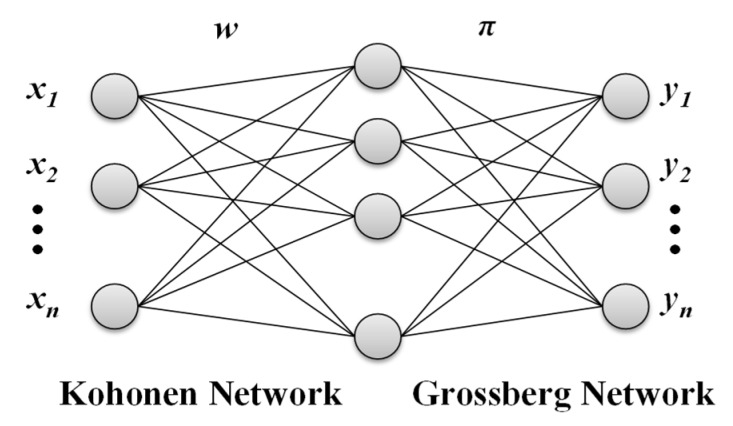
CPN architecture.

CPN area mixed network that combine supervised and unsupervised learning. CPN, which were proposed by Hecht-Nielson [20], act as a statistically optimal self-programming lookup table. A CPN consists of an input layer, a competitive layer, and an output layer, as shown in Figure 2. The input and competitive layers form the Kohonen network, whereas the competitive and output layers form the Grossberg network. It adopts the winner-take-all learning rule in the training process. Only one neuron in the competitive layer can be the winner. The CPN model has a training pair (*X*, *Y*). This pair contains the input vector *x*_1…*n*_ and output vector *y*_1…*n*_. *W* is a weight matrix between the input layer and competitive layer. *π* is a weight matrix between the competitive layer and output layer. In the competitive layer, neurons are generated dynamically rather than located in advance. The Kohonen network is an unsupervised learning network that computes the Euclidean distance between the input vector and weights of each competitive layer node (hidden node), and determines the winner node with the shortest distance. The Grossberg network then uses the winner node’s weights as outputs and adjusts the output weights according to supervised learning. In this study, the CPN model was used to identify the task behavior and predict the corresponding frequency according to the memory access rates (MARs, as defined in [8]) and the performance that a user expects. Thus, the predicted frequency can be applied to the CPU during the computation time.

### 3.2. CPN Learning

The CPN training process has two levels: unsupervised and supervised learning. Unsupervised learning computes the similarity or distance from an input vector *X* to the weight matrix *W*, as mentioned previously. If the distance is acceptable, then the old weight $w_{old}^{j}$ is changed as follows:

In (2), *α* is a learning speed between 0 and 1. *X*(*t*)*Xt* is an input vector at time interval *t*. $w_{old}^{j}$ is the chosen node’s old weight. If the distance is not acceptable, then a new node is added in the competitive layer and assigned the weight $w_{old}^{j}\;$ woldj to the new node’s weight.

(2) $$w_{new}^{j}=w_{old}^{j}+\alpha ⎣X\left(t\right)-w_{old}^{j}⎦.$$

After the CPN finishes unsupervised learning, the training starts supervised learning. The goal of this supervised learning process is to obtain mapping for input objects to desired outputs, given training sets that consist of input and output pairs. If the chosen node in the competitive layer is present, then it changes according to the following formula:

In (3), *β* is a learning speed between 0 and 1. *y*(*t*) is the supervised output vector at time interval *t*. $\pi_{old}^{j}$ *i*s the old weight in the output layer. If the chosen node in the competitive layer does not exist, then a new weight is added to a new hidden node (4).

(3) $$\pi_{new}^{j}=\beta \left(y\left(t\right)-\pi_{old}^{j}\right),$$

(4) $$\pi^{new}=y\left(t\right).$$

### 3.3. CPN Algorithm

The purpose of the CPN training algorithm is to obtain the mapping from *x*(*t*) to *y*(*t*). The CPN learning algorithm is displayed in Table 1. The algorithm is divided into these steps:

(1) At the initial phase, the weight *W* and *π* are all set at zero. Δ is the acceptable distance. *N* is the number of hidden nodes and is initialized as zero. *t* is the learning counter.
(2) First, the initial node is generated in the hidden layer. For all training data, the CPN learning algorithm compares the Euclidean distances between *x*(*t*) and *W*.
(3) Second, the minimum distance between *x*(*t*) and *W* is found. After computing all the distances, the minimum distance *D* can be identified. If *D* is higher than Δ, then the CPN learning algorithm builds the new node in the hidden layer. If *D* is lower than Δ, then the minimum distance node *j* is chosen.
(4) The final step is to adjust the weight $w^{j}$ and $\pi^{j}$ according to the input *x*(*t*) and output *y*(*t*). If the training process is not finished, then it reverts to reading input training data.

**Figure 3:**
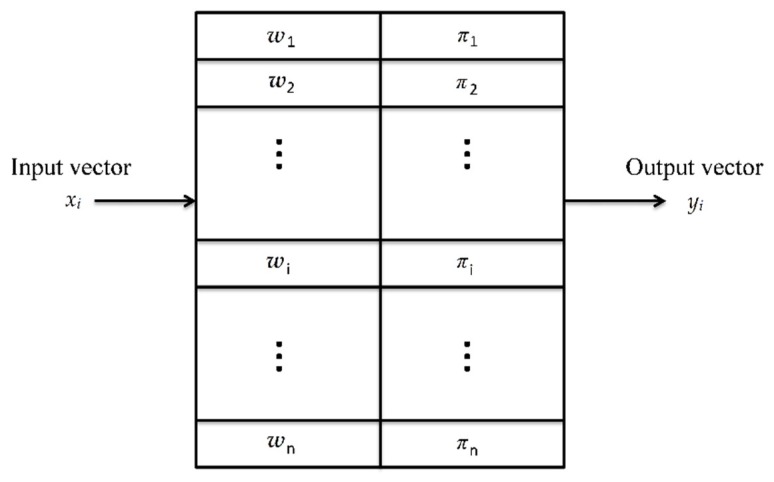
The CPN functions as an adaptive lookup table.

Training process of CPN is the construct process of topology, as the acceptable distance Δ set lower, the nodes in the hidden layer increase, hyperparameters grows. The training time requirement is also affected from the setting of acceptable distance, Δ. After the training process is complete, the network functions as a lookup table. Figure 3 presents an example of the lookup table. The input vector *x* is compared with the weight matrix *W* in the Kohonen network to identify the nearest weight *w_i_*. The output weight vector *π_i_* associated with *w_i_* is selected as the output vector *y_i_*. This is highly similar to the function of a nearest-match lookup table.

## 4. Learning-Based DVFS Schemes

In this section, the proposed learning-based DVFS schemes for single-core and multicore processors are introduced and explained in the following subsections.

### 4.1. Single-Core CPN-DVFS Scheme

Previous studies [6,7,8] have indicated that the CPU frequency appears in a close relationship with both CPU and memory utilization. Hence, we employed the memory access rates (MAR) indicator in this study to represent the memory access property of a program. The definition of MAR is displayed as

(5) $$\mathrm{MAR}=\frac{N_{cache\;miss}}{N_{instr\;exec}}.$$

**Figure 4:**
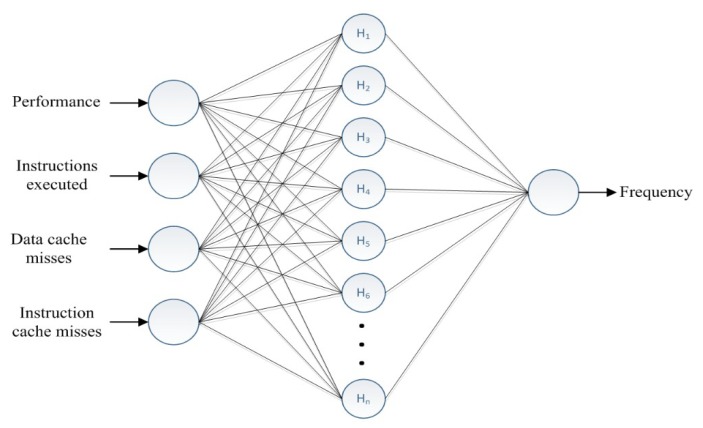
CPN-DVFS architecture.

These values obtained from the performance monitor unit (PMU) are applied to compute the data of MAR and system performance to build up the relation between the CPU frequency and the PMU data. In this study, we applied several benchmarks from MiBench [3] in the experiments and collected five types of features: system performance, numbers of instructions executed, data cache misses, instruction cache misses, and CPU frequencies. Table 2 shows part of the instruction counts of MiBench [3], this study applied 80% of the instructions of the following seven benchmark programs from it including basicmath, bitcount, susan, jpeg, mad, sha, and fast Fourier transform (FFT) for training. By the native Linux system performance analyze toolkit, Perf, these five types of features can be collected in our training and testing experiments. Figure 4 illustrates the topology of the CPNs used in the proposed learning-based DVFS algorithm. The input data are the performance and number of executed instructions as well as the data cache miss and instruction cache miss rates. The appropriate CPU frequency is thus determined as the output result. During the training process discussed in Section 3, mapping is obtained from the input data to generate the desired output results. Therefore, we applied different frequencies in this study for the benchmark programs and collected information about the task behavior for each time interval by the PMU.

As shown in Figure 4, the inputs are instructions executed, data cache misses, system performance, and instruction cache misses. The output result is a frequency. On the basis of the characteristics of input samples and acceptable distance Δ, the hidden nodes *H_n_* are dynamically generated.

The system performance is difficult to evaluate when the system is running. Numerous studies have used the execution time as an indicator while measuring the performance, which is defined as [6]

(6) $$\mathrm{Performance}=1-{\mathrm{PF}}_{\mathrm{loss}}=1-\left(\frac{T_{f_{n}}-T_{f_{\max}}}{T_{f_{\max}}}\right),$$

where PF_loss_ is the performance loss, *f*_max_ is the maximum frequency, and *f_n_* is a frequency lower than *f*_max_. *T_fn_* and *T_f_*_max_ are the task execution times at frequencies of *f_n_* and *f*_max_, respectively. However, the samples in this study were collected during a fixed-length period; thus, the mere execution time could not be relied onto evaluate the performance. By definition, the highest performance occurs at the highest frequency. The execution of a running program can be divided into two parts. The first part involves the time allocated in ideal CPU operations. The second part pertains to the time allocated in external memory accesses, which are determined by the number of cache misses and are closely related to the behavior of the running program. Therefore, a scoreboard method is used to compute the performance score

(7) $$\mu =\rho \cdot N_{instr}+\sigma \cdot N_{mem},$$

*ρ* is a weight of the CPU instruction executed (*N_instr_*), and *σ* represents the factor of the memory access scores (*N_mem_*). *μ* is the computation performance factor. Assume the maximum value of *μ* is 100 at the highest frequency. The highest frequency data can be used to calculate *ρ* and σ scores. Thus, *ρ* and *σ* can be calculated by solving linear equations. However, the highest frequency data have many pairs. Therefore, the results of all *ρ* and *σ* scores calculated are averaged. The performance score *μ* can be calculated at other frequencies according to the CPU and memory operation. Table 3 lists examples for the PXA270 platform. The test samples are obtained from the selected benchmarks of Mibench (as listed in Table 2). Notably, at a 312-MHz frequency, the performance score is 100. This is because of the high memory access time. It is an effective point for savings power without any other performance loss. For a 416-MHz frequency, the performance score is 63 because of a shorter memory access time. It is a CPU-bound interval that loses a lot of performance when the frequency is reduced to 416 MHz.

After training the CPN model, users can choose an acceptable performance between 0% and 100% according to different requirements. In the experiments, the CPN model was used to identify task behavior and to predict the corresponding frequency according to the MAR and performance that a user has chosen. Thus, the predicted frequency could be applied to the CPU during the computation time.

Our previous study [7] determined that the lowest energy consumption generally appears at an operating speed other than the highest and lowest frequencies. Hence, setting the lowest CPU frequency does not entail the lowest energy consumption. The next section demonstrates that the lowest energy consumption generally appears when the performance score is set at approximately 70% in our experiment platform. Table 4 shows the CPN-DVFS algorithm. At the initial stage, the CPN-DVFS resets the PMU counter registers and obtains the weight vector *w_j_* and *π_j_* from the training phase. In every execution interval, the algorithm stops, obtains the input vector *x*(*t*) from the PMU, and identifies the minimum distance between *x*(*t*) and *W*. The ideal frequency is the corresponding output *π_j_*.

### 4.2. Multi-Core CPN-DVFS Scheme

To adapt the proposed approach to modern computing platforms, we extended the learning-directed DVFS algorithm to multicore processor systems. In this study, to train the CPN-DVFS on the Nvidia Tegra K1 multicore platform, we used a single core and three other cores offline to run the benchmarks and determine the number of cache misses and instructions executed through different frequencies. The NVIDIA JETSON Tegra K1(TK1) platform supports the adjustment of the voltage and frequency scales from 51,000 to 2,320,500 kHz and voltage between 760 and 1030 mV. Therefore, the performance scores can be determined using (7) for adapting the proposed CPN-DVFS on the TK1 platform. In multi-core experiments, we also applied the selected benchmarks from MiBench [3] as listed in Table 2 in the experiments, and four types of features were collected regarding the CPN-DVFS training, including performance scores, numbers of instructions executed, cache misses, and CPU frequencies. Thereafter, we used the collected data to train the CPN-DVFS model, as displayed in Figure 4.

**Figure 5:**
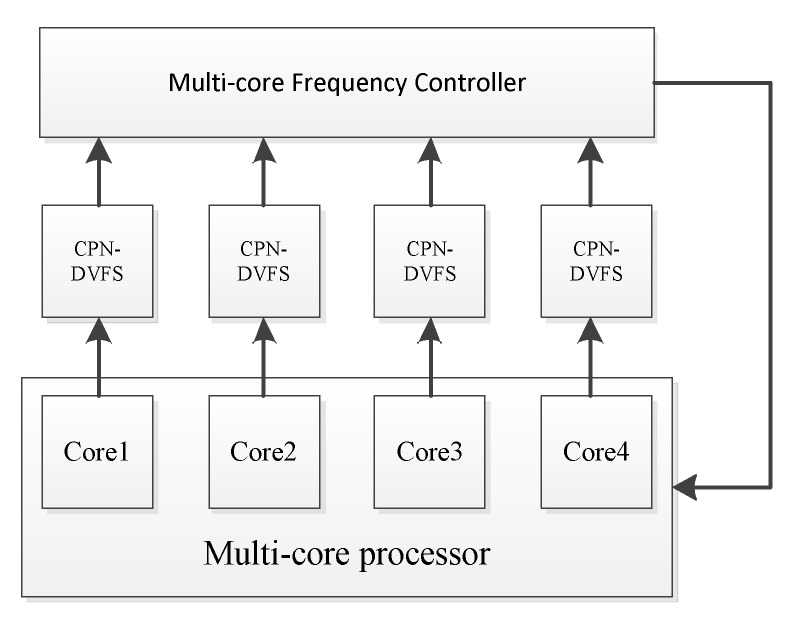
CPN-DVFS for a multicore system.

**Figure 6:**
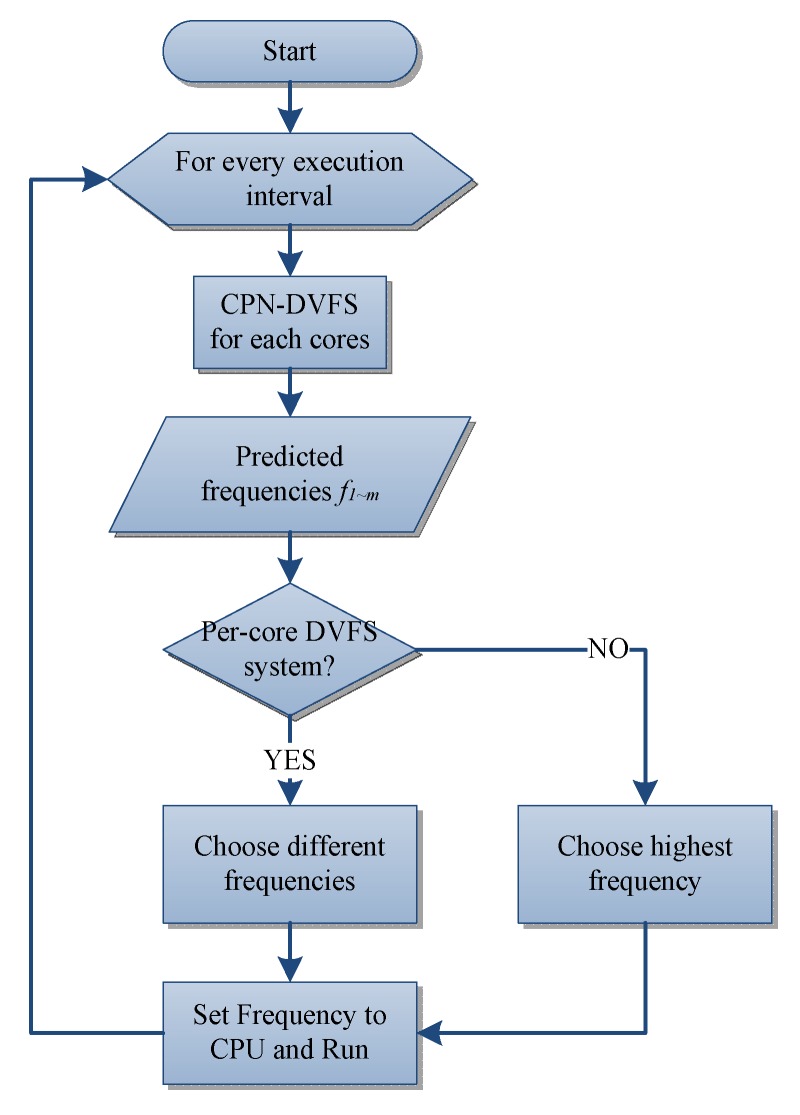
Multicore frequency controller flow chart.

The learning-directed DVFS algorithm for multicore systems is displayed in Figure 5. Each core has its own CPN-DVFS instance to predict the suitable voltage and frequency setting under a running time. In addition, the proposed multicore frequency controller is used to coordinate the dynamic frequency and voltage adjustment according to each CPN-DVFS prediction result. For multicore systems, not all of the individual processor cores can adjust their frequency and voltage asynchronously. A key element of the multicore DVFS technique is the use of one or more voltage regulators that deliver appropriate power consumption to the corresponding circuits. As shown in Figure 6, the multicore frequency controller chooses the CPU frequencies predicted from each CPN-DVFS instance according to the type of multicore DVFS technique. If the target processor supports the per-core DVFS, then each of the individual cores obtains a different appropriate frequency and voltage setting according to the CPN-DVFS prediction results. If the target processor supports only the per-chip DVFS, then the multicore frequency controller chooses the highest frequency among the frequencies determined by the DVFS results of all cores to maintain the system performance.

## 5. Implementation and Measurement

In the Linux kernel, the CPUfreq subsystem provides a modularized interface for managing the CPU frequencies. In Linux, the policy manager for DVFS is called a governor, which controls the CPU frequency through the CPUfreq interface.

**Figure 7:**
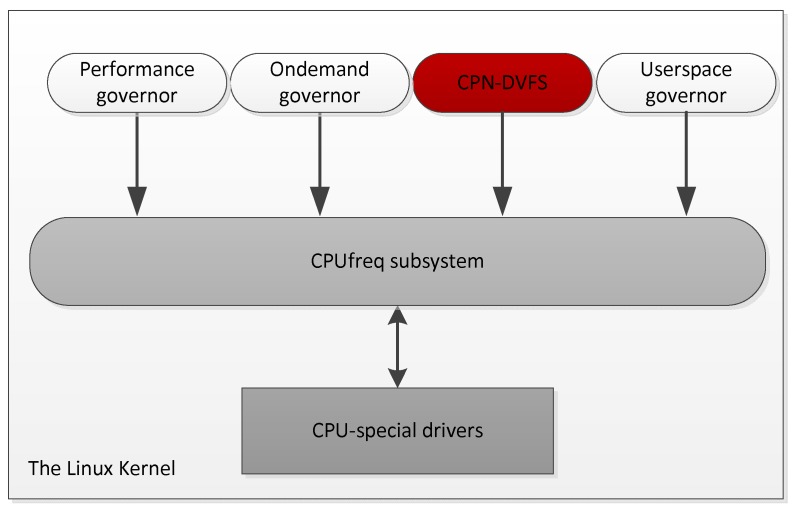
The infrastructure of CPUfreq.

Figure 7 illustrates the CPUfreq infrastructure, in which the CPUfreq subsystem decouples the driver of the CPU-specific hardware from the policies. Numerous kernel-level governors have been supported by Linux for CPU frequency management including the ‘Performance’, ‘Ondemand’, and ‘Userspace’ governors. The Performance governor uses the highest frequency at all time, and the Ondemand governor manages the frequency according to the CPU utilization. Linux also provides the Userspace governor that the user-level governors can control the frequency through the sysfs interface. Among the governors, Ondemand is normally used as the default DVFS mechanism in Linux. In this study, we implemented the proposed DVFS mechanism, called the CPN-DVFS governor. This governor can access the performance counters that realized as the PMU, and set the CPU frequency directly through the Linux CPUfreq subsystem, which is adopted as an additional CPU frequency management without interference with the operation behavior of the native Linux CPUfreq module. The system information such as instructions executed, data cache misses, instruction cache misses and the workloads can be obtained from the performance counters and the OS scheduler respectively operated the CPN-DVFS for each 100 ms time intervals during the run-time execution of the programs. For training the CPN model in Table 1, we used the 80% of instruction counts of the selected seven benchmarks from MiBench benchmarks as listed in Table 2 to run under each CPU frequency and collected information about the task behavior for each time interval.

**Figure 8:**
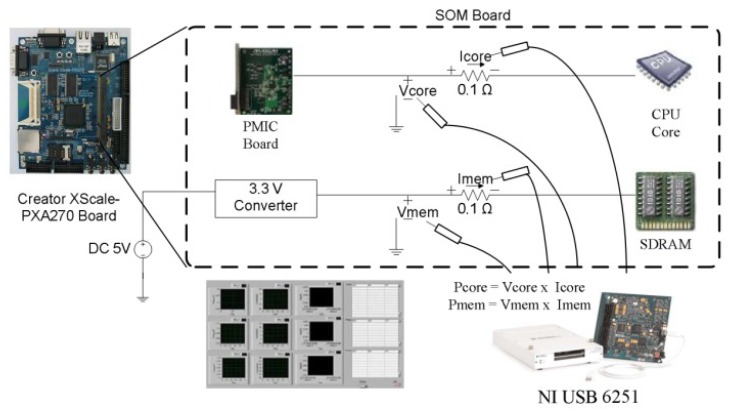
Measurements of the Creator PXA270 components.

Actual energy data were collected using a high-performance DAQ. This DAQ was used to collect the measured data at a rate of 1000 samples/s. The experiment measured the voltages and currents of the CPU and synchronous dynamic random-access memory (SDRAM) to compute the power consumption on a PXA270 platform. Other DVFS methods, including the Linux Ondemand governor, Performance governor, and other learning DVFS governors were also measured for comparative performance evaluation. Figure 8 illustrates the configuration of our measurement environment. All power consumption data were collected from real embedded and mobile platforms. For the TK1 platform, because the energy consumption of CPU and memory could not be separately obtained from the TK1 platform (in contrast to the PXA270 platform experiment), the overall energy consumption of the TK1 platform was measured in this study.

## 6. Experiments

This section reports the experimental results of the proposed learning-based DVFS schemes and comparative results with the standard Linux DVFS implementations for single-core and multicore systems.

### 6.1. Single-Core Platforms

The experiments were performed on a real platform, the Creator PXA270 development board, on which the Linux kernel 2.6.25 was ported. The supported frequencies are listed in Table 5. Two low-resistance sense resistors were used to measure the voltages and currents of the components such as the CPU and SDRAM. The MAXIM 1586B PMIC board (Maxim Integrated, San Jose, CA, USA) was used to support the dynamic voltage adjustment. When the frequency was changed, the corresponding voltage was changed accordingly. The DVFS mechanisms, including the Linux Ondemand governor [22], Performance governor, memory-aware dynamic voltage and frequency scaling (MA-DVFS) [7], and off-chip latency driven Dynamic voltage and frequency scaling (OL-DVFS) [13], were also implemented and measured to compare the experimental results.

**Figure 9:**
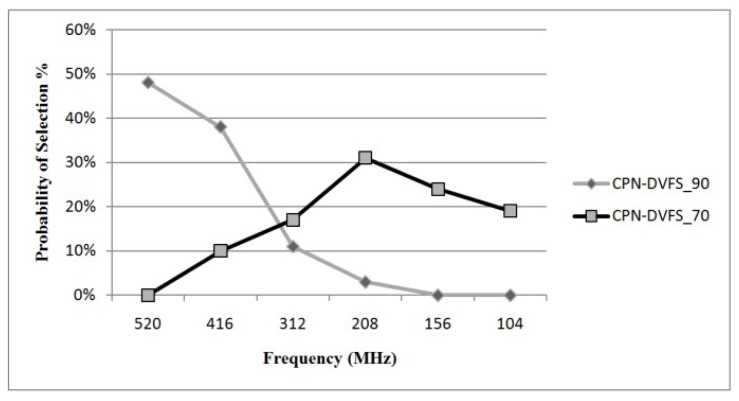
Selection frequency for different levels of required performance.

**Figure 10:**
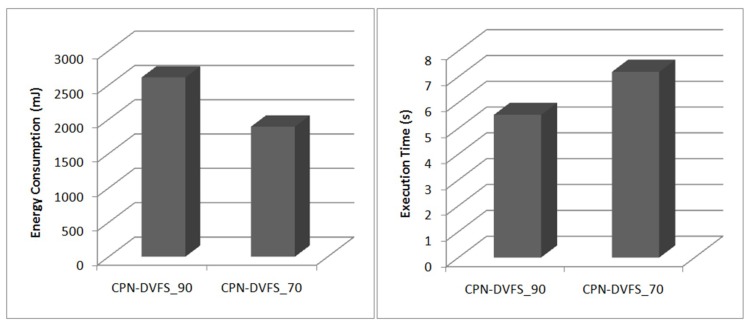
Energy consumption and execution time of gzip_b.

In the experiments for the proposed CPN-DVFS algorithm, the required performance was set at 70% and 90%. The required performance set at 70% is called CPN-DVFS_70, and the required performance set at 90% is called CPN-DVFS_90. Figure 9 depicts the probability that the frequency has been selected for CPN-DVFS_90 and CPN-DVFS_70 on the PXA270 development board. According to the recorded information, CPN-DVFS_90 selected 520MHz 48% of the time when the programs were tested and 39% of the time for 416 MHz. However, CPN-DVFS_70 selected frequencies of 312 MHz and below approximately 73% of the time. This demonstrates that the performance set at 70% is more aggressive than that set at 90% regarding energy savings. Figure 10 shows the energy consumption and execution time of gzip_b in MiBench with the performance set at 70% and 90%. The CPN-DVFS_70 saves more energy than CPN-DVFS_90 does. However, more execution time is also induced forCPN-DVFS_70.

**Figure 11:**
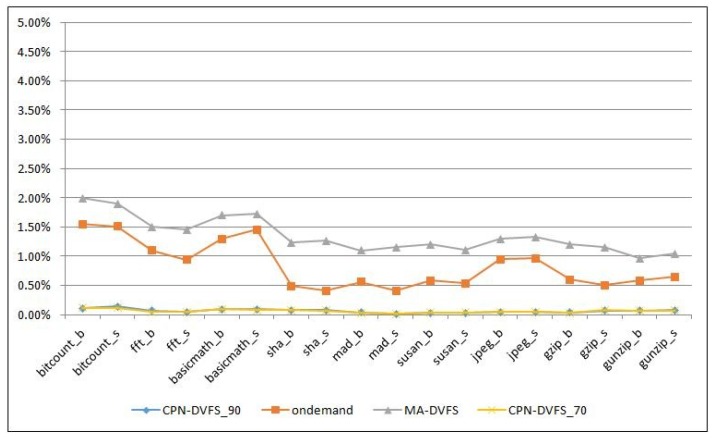
The overhead of the DVFS algorithm.

The DVFS implementation generally has two primary overhead items in the operating systems: DVFS algorithm execution intervals and DVFS computational overhead [3]. One advantage of the CPN is that it is an extremely lightweight neural network technique. To measure the overhead introduced by the DVFS algorithm, a type of Linux kernel information, jiffies, is used. The jiffies records the execution time before and after the DVFS algorithm. In this study, we observed that the DVFS scheme overhead was very low for the MiBench benchmark. The overhead result is shown in Figure 11; in mad_s, the proposed DVFS scheme only costs 0.02% overhead within the entire execution period. The least favorable result is bitcount_s, in which the DVFS overhead cost is approximately 0.12% of the execution period. However, the overhead of the MA-DVFS and Linux Ondemand governor are 0.4% to 2%. Therefore, the presented CPN-DVFS algorithm is relatively lightweight.

**Figure 12:**
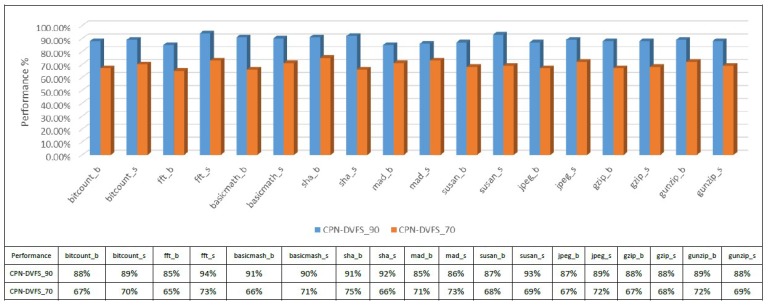
Performance comparison of the CPN-DVFS algorithm for different user preferences.

Figure 12 presents the CPN-DVFS performance in MiBench benchmarks calculated in real execution time to confirm the accuracy of the expected performance configuration (i.e., the value set by the performance factor). For example, the performance of the proposed CPN-DVFS_90 algorithm is approximately 90%. A similar situation also occurs in the cases of CPN-DVFS_70. The error rates of the proposed CPN-DVFS performance are all lower than 6%.

**Figure 13:**
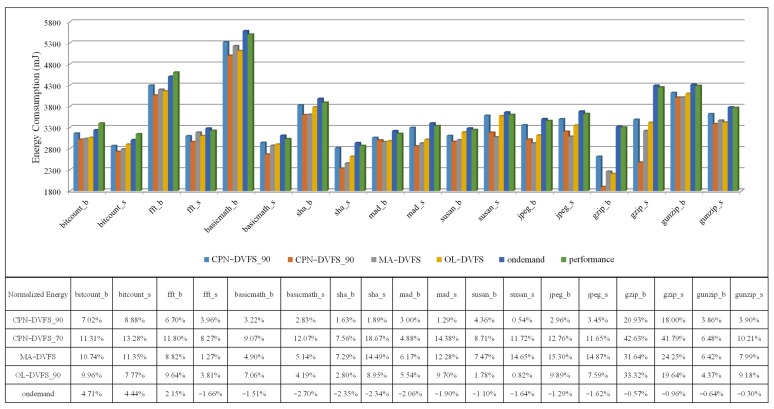
Energy consumption of the MiBench programs.

A comparison of the proposed CPN-DVFS algorithm, the Linux Ondemand governor [22], the Performance governor, the MA-DVFS [7] and OL-DVFS [13] is presented in Figure 13. In the OL-DVFS, it used coarse-grained levels (Low *α*, Med *α*, and High *α*) to control the delay and energy balance. In the implementation, we used Med α to perform the comparative experiments. As depicted in Figure 13, the proposed CPN-DVFS_70 reduced the energy consumption by 4.88% to 42.63%. By comparison, the MA-DVFS reduced the energy consumption by approximately 31.64% at most, whereas the OL-DVFS saved 0.82% to 33.32% of energy consumption. Consequently, the proposed CPN-DVFS_70 demonstrates the most favorable results for energy consumption because CPN-DVFS_70 can tolerate a longer delay. The proposed CPN-DVFS_90 saved 0.54% to 20.93% of energy consumption, yielding superior results to those of the Performance and Ondemand governors. For energy consumption, the CPN-DVFS_90 obtained a result that was slightly inferior to those of the MA-DVFS and OL-DVFS. Because the CPN-DVFS_90 maintained the performance under a loss of less than 10% for higher performance requirements. For a fair comparison between energy and delays, this paper compares the energy delay product (EDP) for the CPN-DVFS_90 in the next figure. The user-set performance that is lower than the CPN-DVFS_70 is also evaluated. Notably, energy consumption began to rise when the user-set performance was lower than 70% of the computing resource. This demonstrates that prolonging the execution time to achieve superior energy savings is inappropriate. Therefore, users should set the CPU performance at higher than 70%.

**Figure 14:**
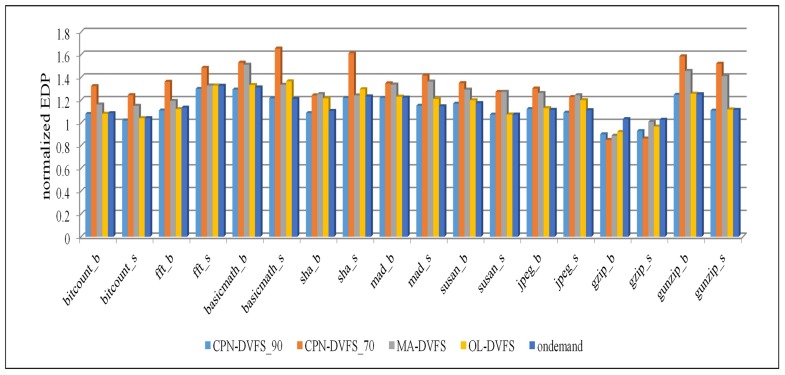
EDP of the MiBench programs.

Figure 14 presents the EDP results of the benchmarks. A lower EDP value generally implies that a greater balance between the performance and energy consumption has been achieved. In this study, the energy consumption and execution time were first normalized to the values of the Performance governor and then multiplied to obtain the EDP values. According to the results, the proposed CPN-DVFS_90 consistently yields EDP outcomes that are more favorable than those of the Linux Ondemand governor, MA-DVFS method, and OL-DVFS. In most benchmarks, the CPN-DVFS_70 obtains, by a slight margin, the least favorable EDP among all governors because CPN-DVFS_70 typically cannot save power by up to 30% when it maintains a performance loss of 30%. These results indicate that the benchmarks might not be suitable for reflecting the performance of the CPN-DVFS_70 because of the low MAR of the benchmarks. However, the CPN-DVFS_70 could obtain a more favorable EDP because it can achieve a significant power consumption reduction in memory-intensive benchmarks. For example, gzip has a higher probability of saving more energy because of its high MAR. Therefore, the CPN-DVFS_70 achieves a more favorable EDP than other governors do in the gzip benchmark. Thus, in summary, if performance and energy savings are considered, then the proposed CPN-DVFS_90 can be the most favorable choice. If energy savings are more crucial than the performance, then the CPN-DVFS_70 can be used to achieve the most favorable power savings results.

### 6.2. Multi-Core Platforms

CMPs have emerged as a key for embedded computing demands. A newer and more powerful platform, TK1 [23], was used for the multicore DVFS experiment. Jetson TK1 is NVIDIA’s embedded development platform featuring a Tegra K1 SOC with a quad core 2.3 GHz ARM Cortex-A15 CPU. The TK1 supports voltage and frequency scales and can be adjusted from 51,000 to 2,320,500 kHz and 760 to 1030 mV. For modern portable and embedded systems, most devices are per-chip DVFS systems that include the newest TK1 platform. For hardware performance counters, the PMU register provided by the ARM Cortex-A15 processor is used [26]. Each core receives its own task execution instructions and memory access counters to predict the frequency by using the CPN-DVFS.

Several benchmarks from MiBench [3] have been adopted for assignment onto each CPU core for evaluating the multitasking performance and power consumption. For a parallel benchmark, ParMiBench [5] was also chosen in the experiments. In Table 6, MiBench1 and MiBench2 represent combinations of various MiBench benchmarks. MiBench3 runs basicmath, and MiBench4 runs the gzip benchmark for all cores. To ignore the load balancing from MiBench1 to MiBench4, all MiBench benchmarks run infinite loops for 20 s. An open source benchmark, ParMiBench, which is intended for multiprocessor-based embedded systems, was also used in this experiment. For a comparative performance evaluation with this study’s multicore scheme, the eDVFS with 10% decrease in performance [21] and the Bayesian classification-based DVFS [17] were implemented on the NVIDIA Tegra K1platform.

**Figure 15:**
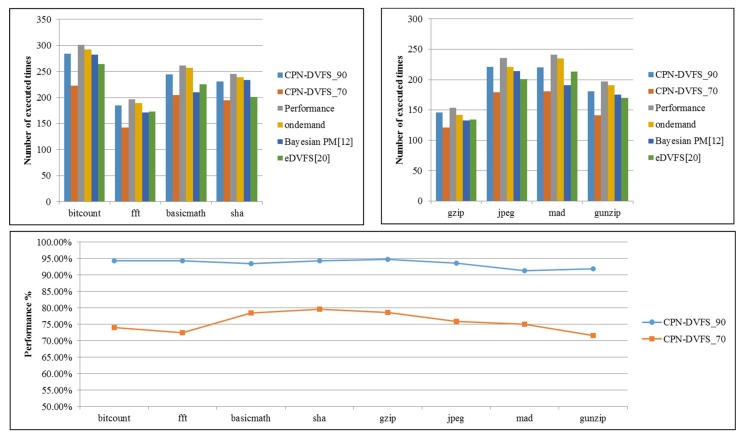
The performance results of MiBench1 and MiBench2.

**Figure 16:**
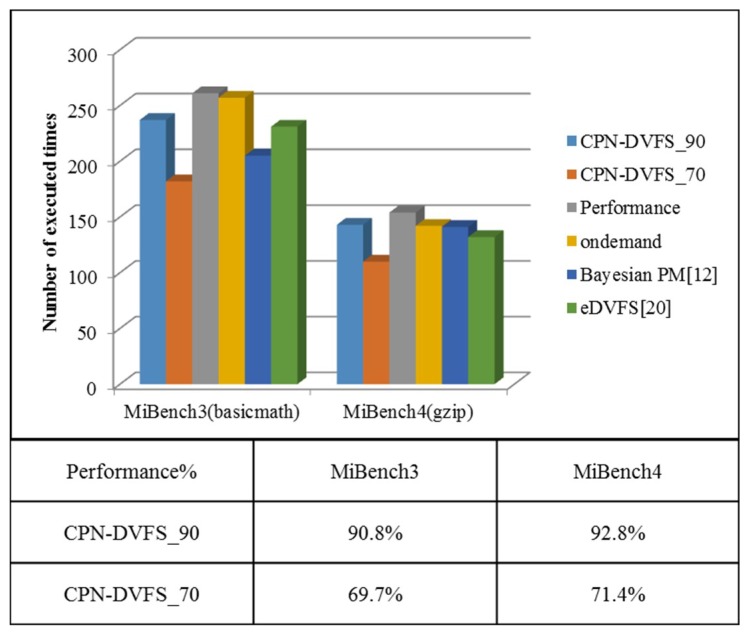
Performance of MiBench3 and MiBench4.

Figure 15 displays the number of executed loops of MiBench1 and MiBench2 on the TK1 platform. The Performance governor consistently ran at the highest frequency, 2,320,500 kHz. The Linux Ondemand governor scales the frequency according to the utilization for each core. The TK1 platform is a per-chip DVFS system. Therefore, the multicore frequency controller uses the highest frequency among the four frequencies determined by the DVFS that are performed on the four cores to preserve the system performance. The Bayesian power management (PM) [17] also uses the multicore frequency controller to determine the final frequency for the per-chip DVFS system. As displayed in Figure 15c, the performance of the CPN-DVFS_90 algorithm is above 90%, and the performance of the CPN-DVFS_70 algorithm is between 70% and 80%. The purpose of this conservative scheme is to prevent losing more performance than the one user expected to lose during the CPU-intensive task. The performance of the proposed CPN-DVFS approaches obtained from MiBench3 and MiBench4 are shown in Figure 16. According to the performance evaluation results, the performance rates of MiBench3 and MiBench4 obtained by the CPN-DVFS_90 are respectively 90.8% and 92.8% regarding the performance governor, and the corresponding performance rates of the CPN-DVFS_70 are 69.1% and 71.4%, respectively. Compared with the performance result of MiBench1 and MiBench2, MiBench3 and MiBench4 were more accurate regarding users’ expected performance configuration. Because MiBench3 and MiBench4 run the same benchmark for all cores, the CPN-DVFS obtain similar program behavior and select similar frequencies for all cores. Therefore, MiBench3 and MiBench4 can obtain results that are closer to the expected performance.

**Figure 17:**
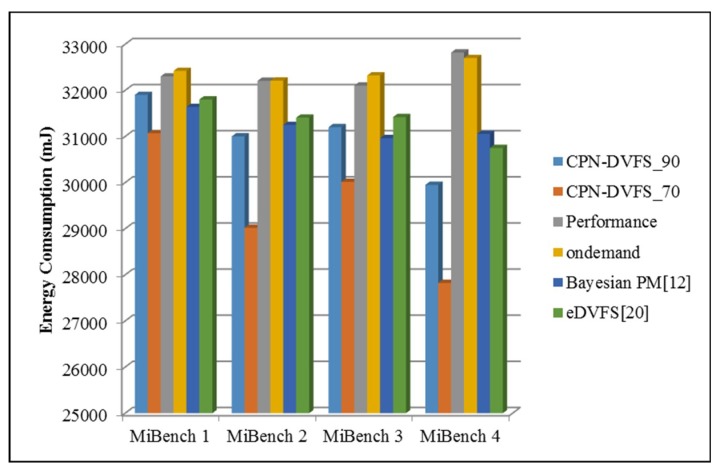
Energy consumption of the MiBench.

The comparative results of energy consumptions between the CPN-DVFS algorithm, Linux Ondemand governor [27], Performance governor, Bayesian PM [14], and eDVFS [28] is provided in Figure 17. The proposed CPN-DVFS_90 saved 2.3% to 8.8% of energy consumption, indicating that its achieved results that were superior to those of the Performance and Ondemand governors. By comparison, the Bayesian PM saved 2.9% to 5.6% and eDVFS saved 2.6% to 6.4% of total energy consumption. The proposed CPN-DVFS_70 reduced energy consumption by 3.9% to 15.4%. CPN-DVFS_70 produces the most favorable results for energy consumption because CPN-DVFS_70 can tolerate a longer delay. In addition, the results of MiBench4 demonstrate that our CPN-DVFS saved the most energy consumption by 15.4% because of the high MAR in the gzip benchmark.

**Figure 18:**
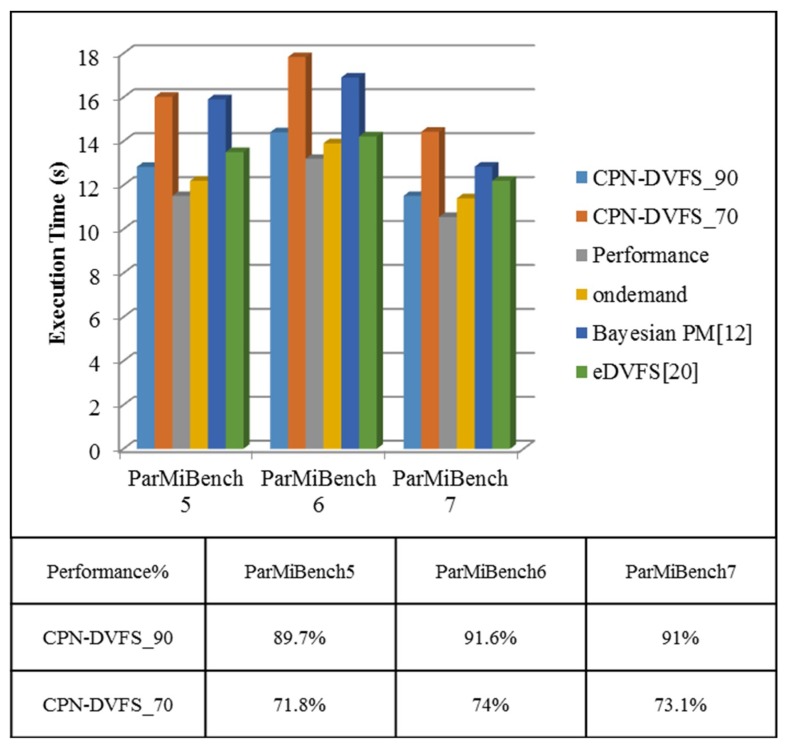
Execution time of the ParMiBench.

**Figure 19:**
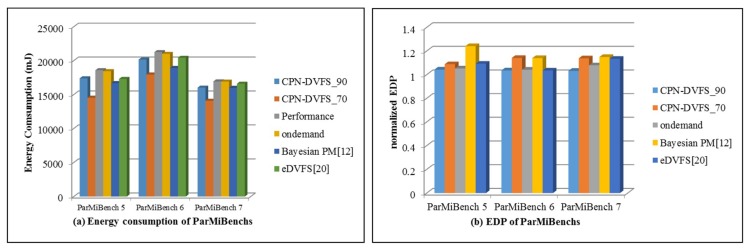
Energy consumption and EDP experimental results of ParMiBenches.

The difference between the benchmarks in ParMiBench and those in MiBench is that the benchmarks in ParMiBench run on multiprocessor-based embedded systems. Figure 18 displays the execution time of ParMiBench. The performance of our CPN-DVFS_70 algorithm is between 71.8% and 74%, and the performance of the CPN-DVFS_90 algorithm is between 89.1% and 91.6%. The performance result is close to the expected performance configuration. Figure 19a presents the energy consumption of ParMiBench. The Bayesian PM saved 5.6% to 11% of total energy consumption. The eDVFS gets 2% to 7% results of saving energy consumption. By contrast, the CPN-DVFS_70 significantly reduces energy consumption by 16% to 22%, and CPN-DVFS_90 saved 5.5% to 6.4% of energy consumption. According to the EDP results of the ParMiBench in Figure 19b, the CPN-DVFS_90 has a superior EDP compared with those of the Linux Ondemand governor, Bayesian PM, and eDVFS method. The proposed CPN-DVFS_70 also obtains superior EDP results compared with those of the Bayesian PM. The multicore experiments in Figure 15, Figure 16 and Figure 18 demonstrate that the proposed learning-directed DVFS method can accurately predict the suitable frequency at the acceptable performance levels set by users. The proposed CPN-DVFS_70 generates outstanding energy savings results, and the CPN-DVFS_90 obtains a more efficient EDP than other comparable governors do regarding the balancing of energy consumption and execution time. In summary, if the performance and energy savings the most effective choice. If energy savings are more crucial than performance, then the CPN-DVFS_70 can be used to achieve the most effective power savings results. Through this method, users can easily achieve effective energy consumption and performance by specifying the allowable performance loss factors.

## 7. Conclusions

This paper proposed a learning-directed DVFS algorithm for single-core and multicore embedded systems. It employs a lightweight CPN to perform the prediction instead of using complicated mathematical models. This study measured the overhead introduced by the DVFS algorithm to prove that the learning-directed DVFS algorithm is relatively lightweight. The proposed approach also provides a mechanism for facilitating control over the trade-off between energy consumption and computation performance. The learning-directed DVFS algorithm was implemented in the Linux operating system on an Intel PXA270 XScale single-core platform and NVIDIA JETSON Tegra K1 multicore platform. In the aforementioned experiments, this study implemented the proposed methods in real embedded devices and collected the actual energy data by using a DAQ to increase the experimental reliability. According to the single-core experiments, the learning-directed DVFS algorithm obtained 5% to 20% energy savings with a 10% performance loss constraint and 9% to 42% energy savings with a 30% performance loss constraint. In the multicore experiments, our approach saved 2.3% to 8.8% energy with a 10% performance loss constraint and 3.9% to 22% energy with a 30% performance loss constraint. On the basis of the experimental results, the proposed CPN-DVFS_70 presents the most outstanding energy savings results, and the CPN-DVFS_90 obtains a favorable EDP for balancing energy consumption and execution time. For modern handheld and mobile devices with a battery power source, the proposed method provides an efficient performance adjustment mechanism to users under various performance requirements. Users can conveniently choose acceptable performance settings according to their demands and application scenarios, as well as extend the endurance of their devices.

Although this paper proposed an efficient learning-directed DVFS technique for single-core and multicore embedded platforms, the proposed technique can be further developed and extended to high-end high-performance computing (HPC) systems. There are several studies [29,30] working on the similar issues for HPC systems, we will proceed to the developments and experiments of HPC systems to validate the performance of the proposed techniques in our future works. As for the dynamic adjustment schemes, this study collected system performance, numbers of instructions executed, data cache misses, and instruction cache misses to predict a proper CPU frequency for the operating system. There is still an additional interesting topic for memory frequency changeable platform, e.g., Nvidia Jetson Tegra series. We will re-design and optimize our DVFS technique to integrate the dynamic adjustment scheme for the memory frequencies to propose a new energy-saving model that is more effective for a memory frequency changeable platform in our future works.

## Figures and Tables

**Table 1:** CPN learning algorithm.

Algorithm: CPN Algorithm	
**Parameters:**α∈[0,1]β∈[0,1]Δ∈[0,∞] **Initialization:** Weight vector *w^j^* and *π^j^* are all zeroed. Weight vector *x*(*t*) and *y*(*t*) are training samples. *t* = 1, *N* = 0.	
1:	Build the first hidden node.
2:	*N* = 1, *t* = *t* + 1
3:	For every training data input do
4:	Choose the nearest node:$D\left(w^{j},x\left(t\right)\right)=\underset{j=1\sim N}{\min}D\left(w^{j},x\left(t\right)\right)$
5:	if D ≦ Δ is true then
6:	Update the weight vector:
7:	$w_{new}^{j}=w_{old}^{j}+\alpha \left[x\left(t\right)-w_{old}^{j}\right]$
8:	$\pi_{new}^{j}=\pi_{old}^{j}+\beta \left[y\left(t\right)-\pi_{old}^{j}\right]$
9:	*t* = *t* + 1
10:	else then
11:	Build the new hidden node.
12:	*N* = *N* + 1, *t* = *t* + 1
13:	end if
14:	end for

**Table 2:** Selected part of MiBench benchmark instruction counts.

Benchmark	Small Instruction Count	Large Instruction Count
basicmath	65,459,080	1,000,000,000
bitcount	49,671,043	384,803,644
susan.corners	1,062,891	586,076,156
susan.edges	1,836,965	732,517,639
susan.smoothing	24,897,492	1,000,000,000
jpeg.decode	6,677,595	990,912,065
jpeg.encode	28,108,471	543,976,667
mad	25,501,771	272,657,564
sha	13,541,298	20,652,916
FFT	52,625,918	143,263,412
FFT.inverse	65,667,015	377,253,252

**Table 3:** PXA270 performance score examples.

CPU Frequency (MHz)	Performance Score (0~100)	Instructions Executed $\left(\times 10^{5}\right)$	Data Cache Miss $\left(\times 10^{3}\right)$	Instruction Cache Miss $\left(\times 10^{2}\right)$
512	100	1514	67	266
512	100	969	1433	847
416	63	1207	116	168
416	82	1236	344	300
312	94	981	692	35
312	100	1035	1471	13
208	60	660	421	16
156	69	250	796	35
104	65	305	689	211

**Table 4:** CPN-DVFS Algorithm.

Algorithm: CPN-DVFS Algorithm	
**Parameters:**performance∈[0,100] **Initialization:** Reset PMU counter registers. Get weight vector *w^j^* and *π^j^* from training phase. pmu_start() for first times calculate.	
1:	For every execution interval do
2:	Pmu_stop()
3:	Choose the nearest node:$D\left(w^{j},x\left(t\right)\right)=\underset{j=1\sim N}{\min}D\left(w^{j},x\left(t\right)\right)$
4:	target_freq = *π^j^*
5:	set_freq(target_freq)
6:	pmu_start()
7:	end for

**Table 5:** Supporting frequency list of the Creator PXA270 platform.

CPU Frequency	CPU Voltage
104 MHz	0.9 V
156 MHz	10 V
208 MHz	1.15 V
312 MHz	1.25 V
416 MHz	1.35 V
520 MHz	1.45 V

**Table 6:** Benchmarks for multicore experiments on TK1.

	*Core1*	*Core2*	*Core3*	*Core4*
MiBench 1	bitcount	fft	basicmath	sha
MiBench 2	gzip	jpeg	mad	gunzip
MiBench 3	basicmath	basicmath	basicmath	basicmath
MiBench 4	gzip	gzip	gzip	gzip
ParMiBench 5	Bitcount			
ParMiBench 6	Basicmath			
ParMiBench 7	Dijkstra			
